# Peptide-mediated display of Tau-derived peptide for construction of microtubule superstructures†

**DOI:** 10.1039/d4cb00290c

**Published:** 2025-03-19

**Authors:** Hiroshi Inaba, Daichi Kageyama, Soei Watari, Mahoko Tateishi, Akira Kakugo, Kazunori Matsuura

**Affiliations:** a Department of Chemistry and Biotechnology, Graduate School of Engineering, Tottori University Tottori 680-8552 Japan hinaba@tottori-u.ac.jp ma2ra-k@tottori-u.ac.jp; b Center for Research on Green Sustainable Chemistry, Tottori University Tottori 680-8552 Japan; c Department of Physics, Graduate School of Science, Kyoto University Oiwake-cho, Kitashirakawa, Sakyo-ku Kyoto 606-8502 Japan

## Abstract

Microtubules are major cytoskeletons involved in various cellular functions, such as regulating cell shape and division and cargo transport *via* motor proteins. In addition to widely studied singlet microtubules, complex microtubule superstructures, including doublets and bundles, provide unique mechanical and functional properties *in vivo*. However, a method to construct such superstructures *in vitro* remains unresolved. This study presents a peptide-based approach for constructing microtubule superstructures by displaying Tau-derived peptides (TP) on the outer surface of microtubules using KA7 peptides as binding units. The KA7-connected TP (KA7–TP) bound to the *C*-terminal tail on the outer surface of microtubules and induced doublets and bundles by recruiting tubulin. Notably, the outer layers of the doublet microtubules generated by KA7–TP dissociated, highlighting the utility of this approach for studying the formation/dissociation mechanisms of microtubule superstructures. The simple peptide-based approach facilitates our understanding of microtubule superstructures and offers new opportunities for applying microtubule superstructures to nanotechnology.

## Abstract 2

Microtubule superstructures including doublet microtubules were constructed by displaying Tau-derived peptide on the surface of microtubules using cationic KA7 peptide.

## Graphical abstract

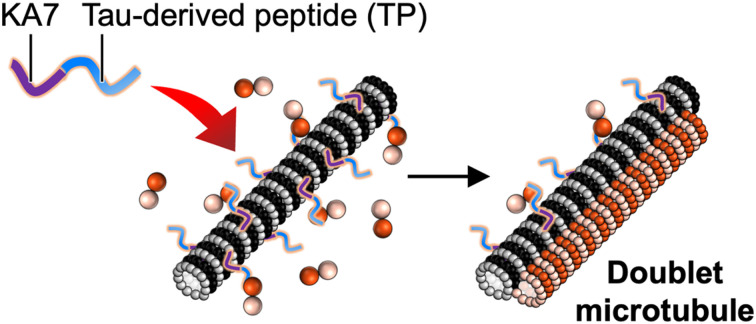

## Introduction

Microtubules are tubular protein assemblies consisting of tubulin dimers and are important cytoskeletons involved in cellular functions, such as controlling cell shape and division, cargo delivery *via* the motility of motor proteins (kinesin and dynein) and as intriguing motile nanomaterials for use in the construction of active matter and molecular robots.^1–4^ In general, microtubules form singlet structures *in vitro* and *in vivo*, which is a single hollow tube composed of protofilaments composed of tubulin. However, there are microtubule superstructures *in vivo* that exhibit unique properties distinct from those of singlet microtubules.^5–14^ These microtubule superstructures include doublet microtubules consisting of a complete singlet microtubule (A-tubule) and an incomplete microtubule (B-tubule) tethered to the A-tubule,^5–8^ multiplets,^9^ branches,^10,11^ asters^12^ and bundles.^13,14^ Microtubule bundles are formed by crosslinking singlet microtubules *via* classical microtubule-associated proteins (MAPs),^15^ which play important roles in axons and dendrites by forming and maintaining neurites.^13,14^ Doublet microtubules obtained from flagella and cilia have intriguing properties, including high stability, mechanical rigidity and intraflagellar transport.^5–8^ Formation of natural doublet microtubules is induced by binding various microtubule inner proteins (MIPs) to the inner surface of microtubules.^5,6,16^ In particular, specific MIPs such as PACRG, FAP20,^17,18^ FAP45, FAP52^19^ and CFAP77^20^ play important roles in anchoring the B-tubule to the outer surface of the A-tubule. Because of their unique properties, constructing these microtubule superstructures *in vitro* is crucial for understanding their functions and use in nanotechnological applications. Although the formation of doublet microtubules by deleting the *C*-terminal tail of tubulin located on the outer surface of microtubules was reported to induce access of free tubulin to microtubules,^21^ a general method for constructing microtubule superstructures remains unknown. If exogenous molecules binding to the outer surface of microtubules can recruit new tubulin, these exogenous molecules would afford significant potential in inducing the formation of microtubule superstructures such as doublets.

We have developed a method for modulating the structures and functions of microtubules based on a Tau-derived peptide (TP).^16^ TP (CGGGKKHVPGGGSVQIVYKPVDL) is a binding unit to the taxol-binding pocket of β-tubulin located on the internal surface of microtubules, which was designed based on a repeat domain of the microtubule-associated protein Tau.^22^ Derivatized TPs have been used to encapsulate various nanomaterials such as proteins^23,24^ and metal nanoparticles^25–27^ inside microtubules.^16^ We also showed previously the formation of microtubule superstructures, including doublets, multiplets, branches, bundles and asters, by the binding of the TP-fused tetrameric protein, Azami-Green (TP–AG).^24^ The binding of TP–AG to the outer surface of microtubules enabled the display of the TP moiety, which bound new tubulin to induce the formation of double microtubules.^24^ Recently, a similar approach using a TP-fused photoswitching tetrameric protein, Dronpa, was used to photocontrol the formation/dissociation of superstructures.^28^ These results show that TP-fused protein scaffolds can be used to present TP on the outer surface of microtubules to induce microtubule superstructures, as achieved by MIPs. Herein, the question arises as to whether microtubule superstructures can be formed if TP is presented on the outer surface of microtubules, even without protein scaffolds.

**Fig. 1:**
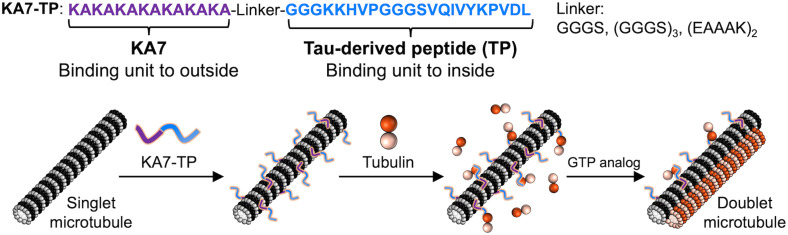
Formation of doublet microtubules by presenting a Tau-derived peptide (TP) on microtubules. Peptides used in this study (top) and schematics of the binding of KA7-connected TP (KA7–TP) to microtubules and formation of doublet microtubules (bottom).

In this report, we developed a peptide-based system for constructing microtubule superstructures by presenting TP on microtubules using the KA7 peptide (KAKAKAKAKAKAKA)^29^ as a binding unit to the outer surface of microtubules (Fig. 1). KA7–connected TP (KA7–TP) bound to the *C*-terminal tail on the outer surface of microtubules and induced formation of bundles and doublets. The simple peptide-based approach should facilitate studies of microtubule superstructures and advance nanotechnology applications.

## Results and discussion

### Design and binding analysis of KA7–TP to microtubules

**Fig. 2:**
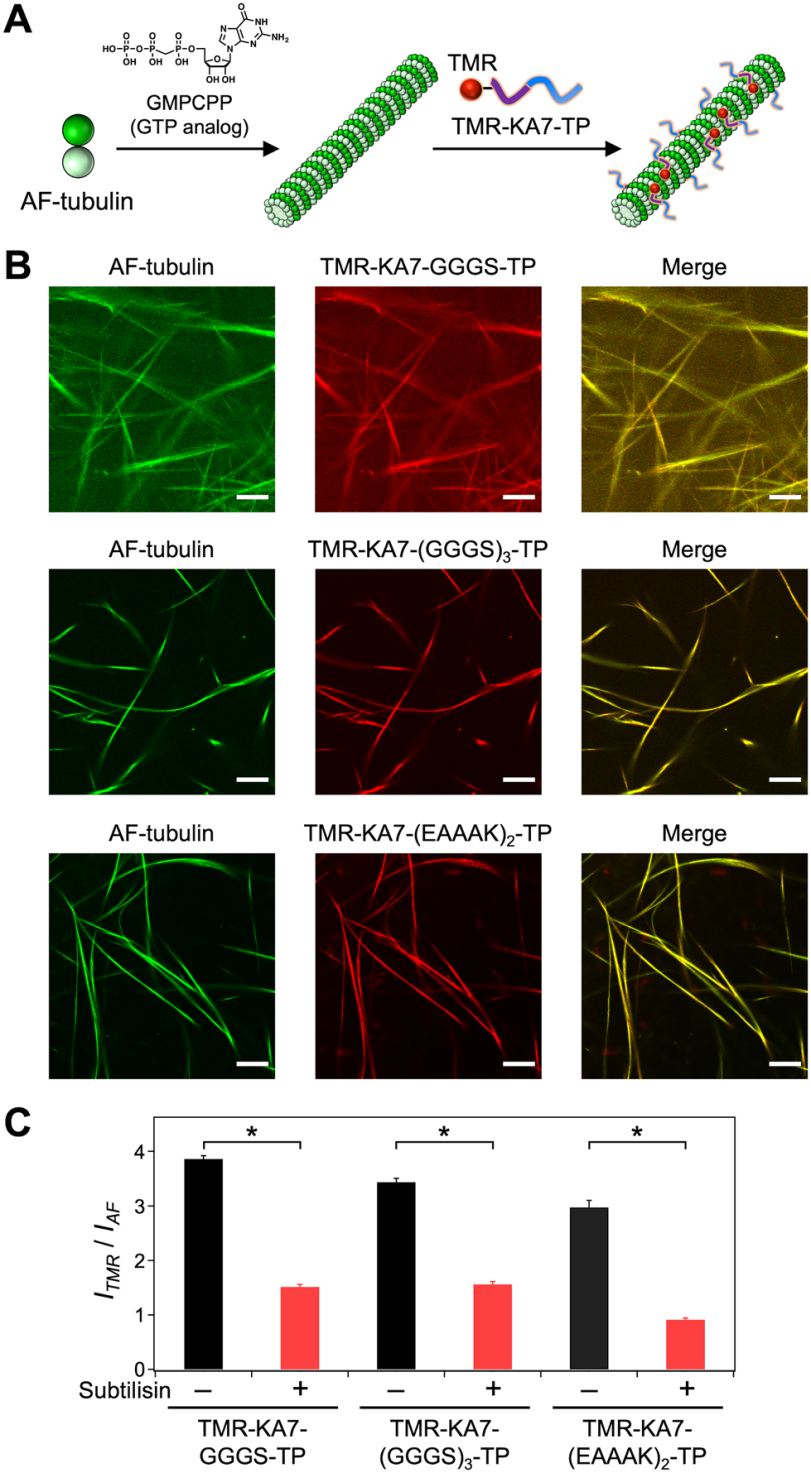
Binding analysis to microtubules. (A) Preparation of Alexa Fluor 488 (AF)-labeled microtubules and the binding of tetramethylrhodamine (TMR)-labeled KA7–TP (TMR–KA7–TP). (B) Confocal laser scanning microscopy (CLSM) images of microtubules bound with TMR–KA7–TP. Preparation concentrations: [tubulin] = 10 μM; [AF-tubulin] = 10 μM; [TMR–KA7–TP] = 80 μM; [GMPCPP] = 0.2 mM. Scale bars, 10 μm. (C) The *I*_TMR_/*I*_AF_ ratio of TMR–KA7–TP-bound microtubules with and without subtilisin treatment showing TMR–KA7–TP fluorescence per microtubule determined from the CLSM images (Fig. S4, ESI†). Error bars represent the standard error of the mean (*N* = 28). *P < 0.0001, two-tailed Student's *t* test. Preparation concentrations: [tubulin] = 13.4 μM; [AF-tubulin] = 3.4 μM; [TMR–KA7–TP] = 24 μM; [subtilisin] = 0.7 μM; [GMPCPP] = 0.14 mM.

KA7 is a positively charged peptide that binds to the negatively charged *C*-termini of tubulins located on the outer surface of microtubules *via* electrostatic interactions.^29^ We designed the tandem peptide KA7–TP consisting of KA7 and TP through different linkers to enable the display of the TP moiety on the outer surface of microtubules (Fig. 1). By using flexible (GGGS) and rigid (EAAAK) linkers,^30^ three KA7–TPs (*i.e.*, KA7–GGGS–TP, KA7–(GGGS)_3_–TP and KA7–(EAAAK)_2_–TP) were designed. The KA7–TPs and red fluorescent tetramethylrhodamine (TMR)-conjugated KA7–TPs (TMR–KA7–TPs) were synthesized by Fmoc-based solid phase chemistry and conjugation of TMR at the *N*-terminus of KA7–TPs on resin. The obtained peptides were purified by reverse-phase high-performance liquid chromatography (RP-HPLC) and confirmed by matrix-assisted laser desorption-ionization time-of-flight mass spectrometry (MALDI-TOF-MS; Fig. S1, ESI†). The binding of TMR–KA7–TPs to Alexa fluor 488 (AF)-labeled microtubules (AF-microtubules) was confirmed by confocal laser scanning microscopy (CLSM). AF-labeled microtubules were prepared by using guanosine-5′-[(α,β)-methyleno]triphosphate (GMPCPP), a slowly hydrolyzable guanosine triphosphate (GTP) analog, to form stable microtubules, and these microtubules were incubated with TMR–KA7–TPs (Fig. 2A). Co-localization of red and green fluorescence confirmed the binding of all TMR–KA7–TPs to AF-labeled microtubules (Fig. 2B). Binding of TMR-labeled KA7 without the TP moiety (TMR–KA7) to AF-microtubules was also observed, indicating the direct binding of the KA7 moiety to the microtubules (Fig. S2A, ESI†). CLSM observations using control samples of only AF-microtubules without TMR-peptides and TMR–KA7-incorporated unlabeled microtubules without AF-tubulin showed no bleed-through under the measurement conditions used (Fig. S2B and C, ESI†). The fluorescence intensity of TMR on the microtubules was stronger for TMR–KA7–(GGGS)_3_–TP and TMR–KA7–(EAAAK)_2_–TP than for TMR–KA7–GGGS–TP (Fig. S3, ESI†), indicating that the longer linkers of TMR–KA7–(GGGS)_3_–TP and TMR–KA7–(EAAAK)_2_–TP facilitated binding to microtubules. Next, the binding sites of TMR–KA7–TPs were evaluated using subtilisin, which digests the negatively charged *C*-terminal tail of tubulin.^21,24,31^ The binding of all TMR–KA7–TPs to the subtilisin-treated microtubules was reduced significantly compared with microtubules that underwent no subtilisin treatment (Fig. 2C and Fig. S4, ESI†). These results showed that the main binding site of these peptides was the *C*-terminal tails of tubulin located on the outer surface of microtubules.

### Formation of microtubule superstructures induced by KA7–TP

**Fig. 3:**
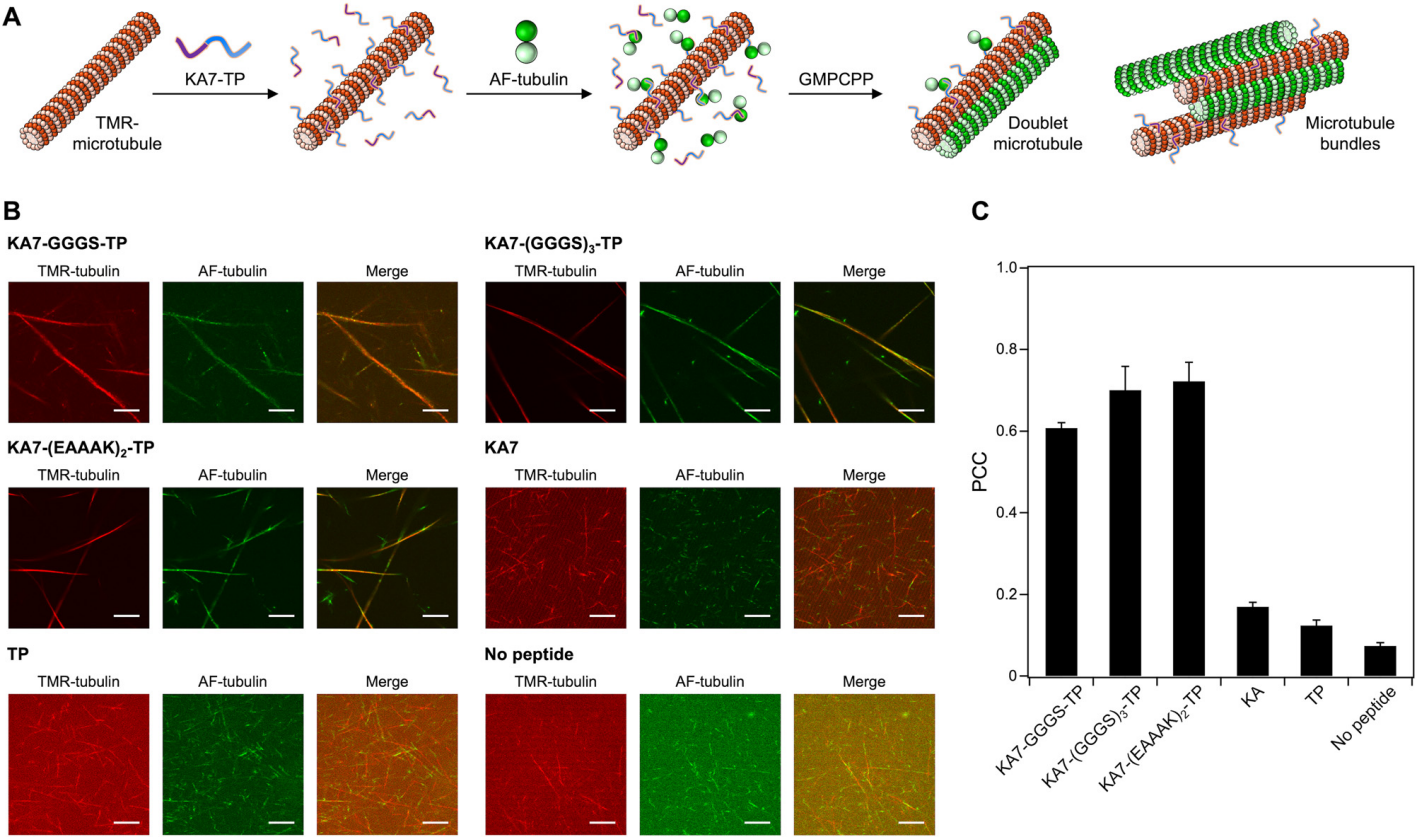
Formation of microtubule superstructures induced by KA7–TP. (A) Schematics of the formation of microtubule superstructures by binding of KA7–TP to the TMR-microtubule (red) and subsequent incubation of AF-tubulin (green) and GMPCPP and (B) the CLSM images. Preparation concentrations: [tubulin] = 2.7 μM; [TMR-tubulin] = 0.9 μM; [AF-tubulin] = 0.36 μM; [KA7–TP] = 100 μM; [GMPCPP] = 0.2 mM. Scale bars, 10 μm. As controls, 100 μM KA or 100 μM TP was used instead of KA7–TP. (C) Pearson's correlation coefficient (PCC) analysis of the CLSM images. Error bars represent the standard error of the mean (*N* = 5).

The ability of KA7–TPs to induce the formation of microtubule superstructures was evaluated using tubulin with different fluorescence colors (red for TMR-tubulin and green for AF-tubulin). TMR-labeled singlet microtubules were prepared and subsequently incubated with KA7–TP, AF-tubulin and GMPCPP for the binding of AF-tubulin to the TP moiety of KA7–TP on TMR-labeled microtubules (TMR-microtubules) to induce the growth of AF-labeled microtubules (Fig. 3A). The co-localization of TMR-tubulin and AF-tubulin fluorescence was observed by using KA7–GGGS–TP, KA7–(GGGS)_3_–TP and KA7–(EAAAK)_2_–TP (Fig. 3B). In contrast, there was less co-localization but growth of AF-microtubules from both ends of TMR-microtubules in the absence of KA7–TPs (Fig. 3B). The growth of AF-microtubules from TMR-microtubules was also observed in the KA7–TP-treated samples; however, the frequency appeared to be low. Moreover, microtubules consisting of only AF-tubulin were rarely observed in the KA7–TP-treated samples. Thus, the formation of microtubule superstructures is presumably preferred under these conditions. The Pearson's correlation coefficient (PCC) was calculated from the images to estimate the degree of co-localization of TMR and AF fluorescence.^32^ The PCCs of the samples using KA7–GGGS–TP (0.61), KA7–(GGGS)_3_–TP (0.70) and KA7–(EAAAK)_2_–TP (0.72) were greater than that determined when no peptide was used (0.07) (Fig. 3C). The co-localization of TMR-tubulin and AF-tubulin fluorescence was much lower than those of KA7–TPs when only KA7 and TP were used (Fig. 3C), indicating the importance of linking KA7 and TP. These results indicate that the formation of AF-labeled microtubules on the surface of TMR-microtubules was induced by KA7–TPs. We used KA7–(GGGS)_3_–TP for further analysis because KA7–(GGGS)_3_–TP and KA7–(EAAAK)_2_–TP showed similar ability in forming microtubule superstructures (Fig. 3).

**Fig. 4:**
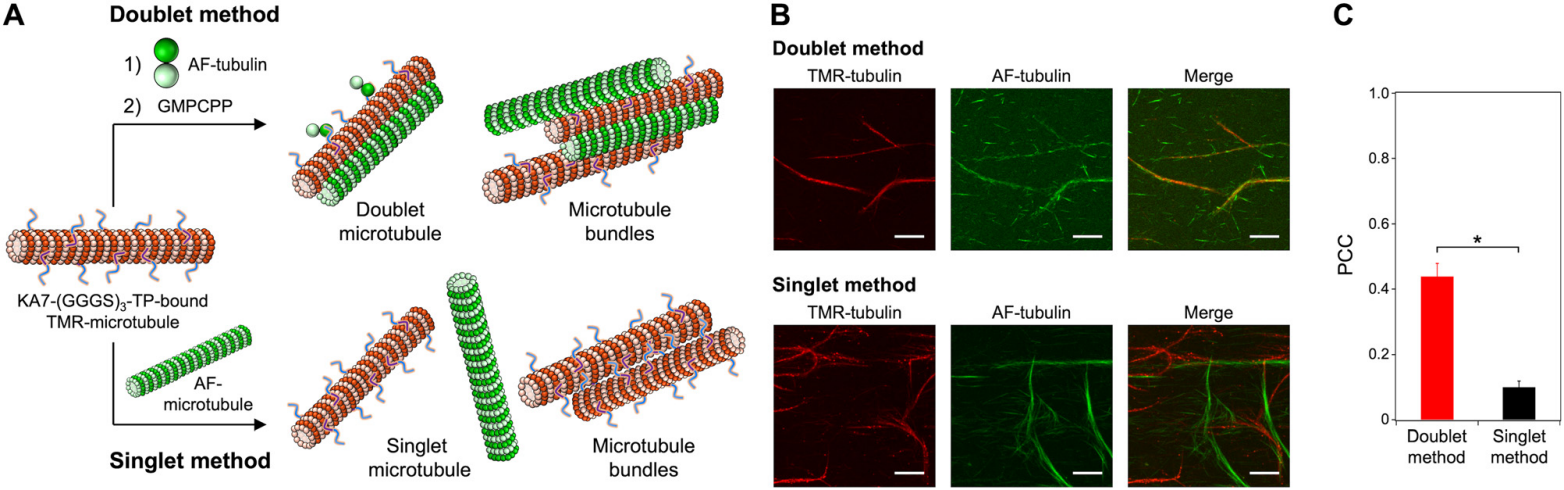
Comparison of the methods for forming microtubule superstructures. (A) Schematics of the formation of doublet microtubules (doublet method) and the mixing of singlet microtubules (singlet method) using KA7–(GGGS)_3_–TP-bound microtubules and (B) the CLSM images. Preparation concentrations: [tubulin] = 2.7 μM; [TMR-tubulin] = 0.9 μM; [AF-tubulin] = 0.36 μM; [KA7–(GGGS)_3_–TP] = 100 μM; [GMPCPP] = 0.2 mM. Scale bars, 10 μm. (C) PCC analysis of the CLSM images. Error bars represent the standard error of the mean (*N* = 7). *P < 0.0001, two-tailed Student's *t*-test.

Increasing the concentration of KA7–(GGGS)_3_–TP caused incremental increases in the co-localization of TMR- and AF-microtubules (Fig. S5, ESI†). The growth of AF-microtubules from TMR-microtubules was dominant when 0–50 μM KA7–(GGGS)_3_–TP was used. The efficient formation of microtubule superstructures was observed by using more than 100 μM KA7–(GGGS)_3_–TP, presumably because of sufficient coverage of TMR-microtubules with KA7–(GGGS)_3_–TP for the accumulation of AF-tubulin and the formation of doublet microtubules. Relatively high concentrations of KA7–(GGGS)_3_–TP were required, presumably because of the moderate binding affinity of the KA7 moiety to microtubules (weak binding at 0.25 μM)^29^ and the TP moiety to tubulin (*K*_d_ = 6.0 μM).^22^ Increasing the KA7–(GGGS)_3_–TP concentration caused a decrease in the densities of the TMR-microtubules and a fluorescence intensity increase of the TMR channel (Fig. S5, ESI†), indicating that the doublet and singlet microtubules accumulated to form bundled microtubules *via* excess KA7–(GGGS)_3_–TP crosslinking (Fig. 3A). Next, different methods for constructing microtubule superstructures were used, and the results were compared. In the “doublet method”, KA7–(GGGS)_3_–TP-bound TMR-microtubules were subsequently incubated with AF-tubulin and GMPCPP to generate doublet microtubules as shown above (Fig. 4A). In the “singlet method”, KA7–(GGGS)_3_–TP-bound TMR-microtubules were incubated with preformed AF-labeled microtubules as a control of the doublet method (Fig. 4A). In the singlet method, doublet microtubules, in principle, cannot form. CLSM images and PCC analysis showed that the co-localization efficiency of TMR- and AF-microtubules was lower using the singlet method compared with that of the doublet method (Fig. 4B and C). The comparison clearly showed that preincubation of AF-tubulin with the KA7–(GGGS)_3_–TP-bound TMR-microtubules and subsequent formation of AF-microtubules is important for generating microtubule superstructures consisting of TMR-microtubules and AF-microtubules. In the doublet method, it is plausible that KA7–(GGGS)_3_–TP bound to the outer surface of microtubules *via* the KA7 moiety and then the exposed TP moiety recruited free tubulin to induce the formation of doublet microtubules. The mechanism was the same as our previous approach using TP-fused tetrameric proteins.^24,28^

### Structures of microtubule superstructures

**Fig. 5:**
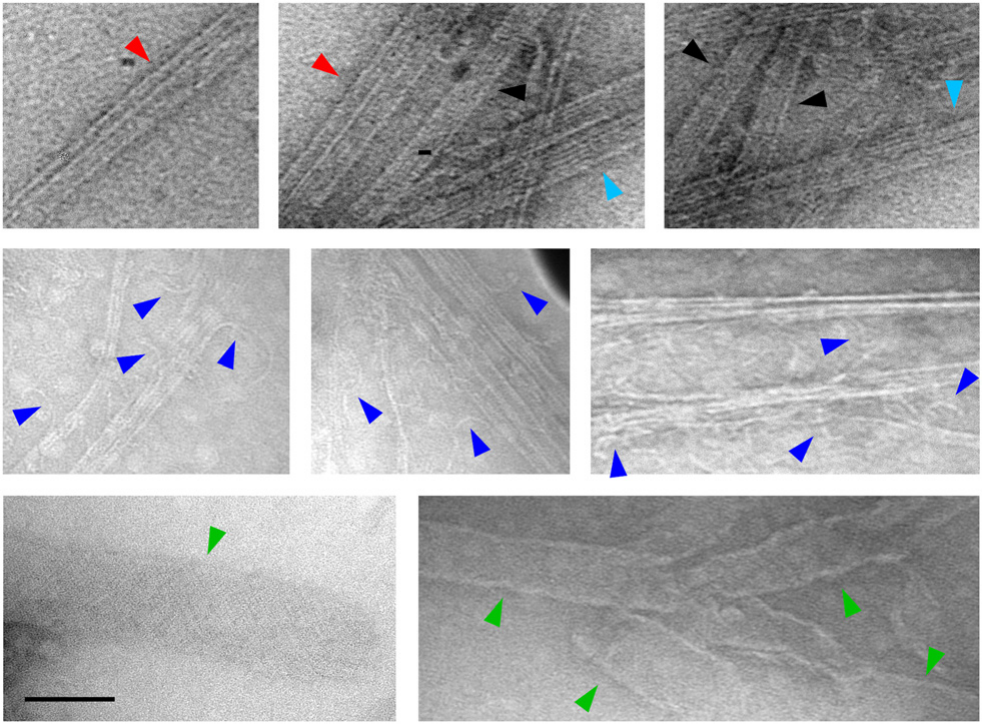
Transmission electron microscopy (TEM) images of microtubule superstructures induced by KA7–(GGGS)_3_–TP using the doublet method. singlet microtubules (black arrowheads), doublet microtubules (red arrowheads), microtubule bundles (cyan arrowheads), peeled structures (blue arrowheads) and sheet structures (green arrowheads) were observed. Preparation concentrations: [tubulin] = 2.7 μM; [TMR-tubulin] = 0.9 μM; [AF-tubulin] = 0.36 μM; [KA7–(GGGS)_3_–TP] = 100 μM; [GMPCPP] = 0.2 mM. Scale bar, 100 nm.

The detailed structures of microtubule superstructures induced by KA7–(GGGS)_3_–TP were observed by negatively staining transmission electron microscopy (TEM). When KA7–(GGGS)_3_–TP was used by the doublet method, not only singlet microtubules (Fig. 5, black arrowheads) but also doublet-like microtubules (Fig. 5, red arrowheads) and bundles of singlet microtubules (Fig. 5, cyan arrowheads) were observed. The doublets and bundles were distinguished by thinner diameters of B-tubules (∼15 nm) of doublets than that of singlet microtubules (∼25 nm) of bundles. In addition to the doublets and bundles, the peeling of microtubule protofilaments (Fig. 5, blue arrowheads) and sheet-like structures (Fig. 5, green arrowheads) were observed. Because B-tubules are generally not as stable compared with A-tubules,^33,34^ it was thought that the additional layers of the microtubules induced by KA7–(GGGS)_3_–TP may detach as peeled structures (intermediate during dissociation) and sheets (complete dissociation) during drying for the TEM measurements. No such peeling or formation of sheets was observed when TP–AG induced the generation of doublet microtubules.^24^ Thus, the doublet microtubules induced by KA7–(GGGS)_3_–TP may not be as stable as those induced by TP–AG. This result is probably because of several differences between KA7–(GGGS)_3_–TP and TP–AG, such as binding affinity, size and flexibility. Only singlet microtubules and bundles of singlet microtubules were observed when KA7–(GGGS)_3_–TP was used in the singlet method or the peptide was not used in the doublet method (Fig. S6, ESI†). These results indicate that treating KA7–(GGGS)_3_–TP in the doublet method was important for forming doublet microtubules, and the results are consistent with the CLSM images in Fig. 4.

### Stability of microtubule superstructures

**Fig. 6:**
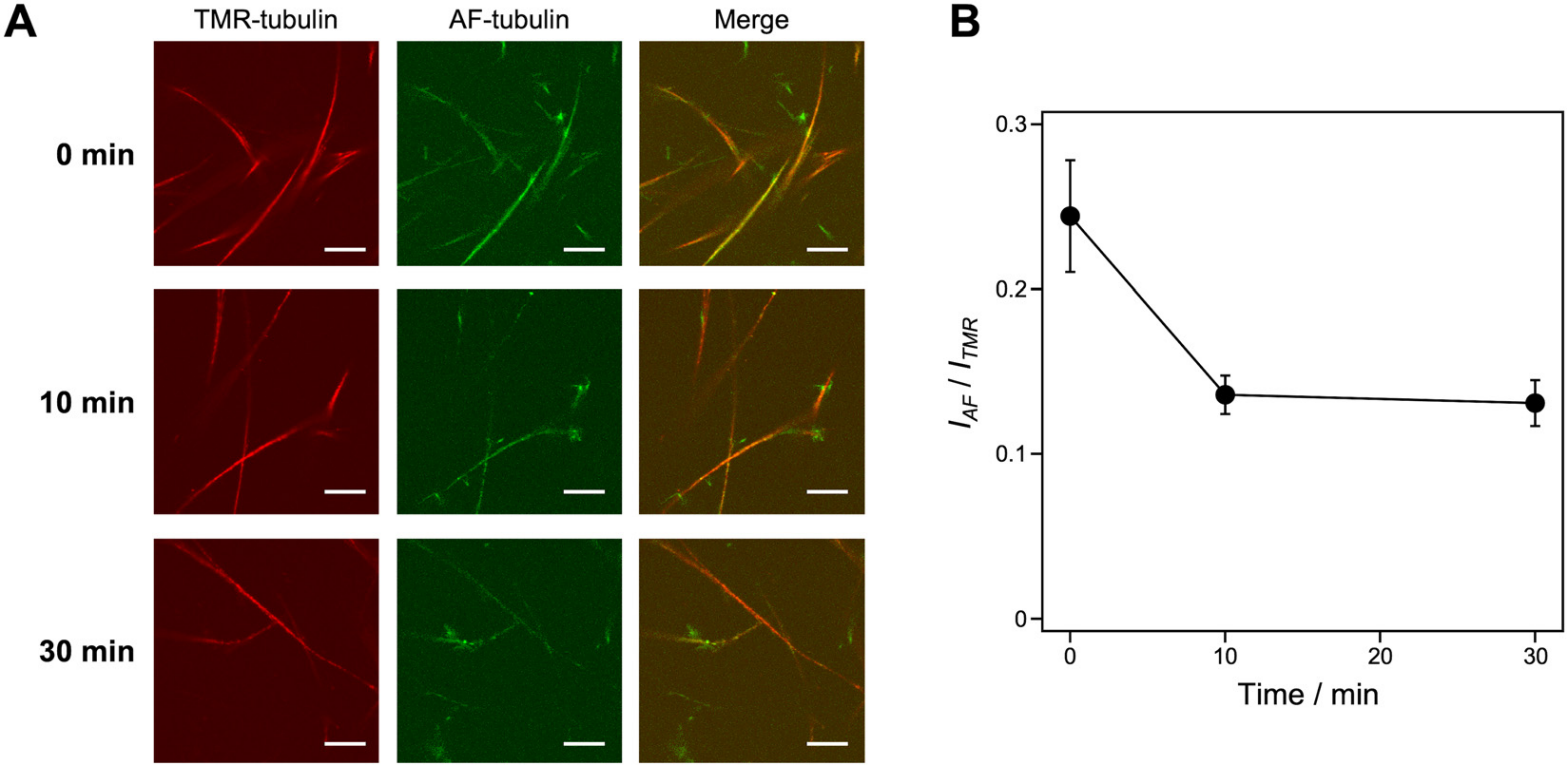
Stability analysis of the microtubule superstructures. (A) CLSM images of microtubule superstructures induced by KA7–(GGGS)_3_–TP using the doublet method, keeping at 4 °C for 0–30 min. Preparation concentrations: [tubulin] = 2.7 μM; [TMR-tubulin] = 0.9 μM; [AF-tubulin] = 0.36 μM; [KA7–(GGGS)_3_–TP] = 100 μM; [GMPCPP] = 0.2 mM. Scale bars, 10 μm. (B) The *I*_AF_/*I*_TMR_ ratio of microtubule superstructures showing AF-microtubule fluorescence per TMR-microtubule determined from the CLSM images. Error bars represent the standard error of the mean (*N* = 12).

The stability of microtubule superstructures induced by KA7–(GGGS)_3_–TP was evaluated by incubating them at a low temperature (4 °C), which induces microtubule depolymerization.^35,36^ The microtubule superstructures were prepared using TMR-microtubules, KA7–(GGGS)_3_–TP, AF-tubulin and GMPCPP in the doublet method (see Fig. 4A). The AF fluorescence on TMR-microtubules decreased upon incubating the microtubule superstructures at 4 °C, whereas TMR fluorescence remained intact (Fig. 6). Because B-tubules are generally less stable than A-tubules, the additional layers consisting of AF-tubulin seem to dissociate gradually from the TMR-microtubules formed by GMPCPP, which are stable at 4 °C. In contrast, the average fluorescence intensity of AF-microtubules per the average fluorescence intensity of TMR-microtubules did not decrease by incubating at 4 °C when prepared by the singlet method (Fig. S7, ESI†). Comparing the doublet and singlet methods showed the different stability of AF- and TMR-microtubules only in the doublet method, indicating the formation of B-tubules consisting of AF-tubulin and A-tubules consisting of TMR-tubulin when using KA7–(GGGS)_3_–TP.

## Discussion

By using tandem peptides consisting of KA7 and TP, the generation of microtubule superstructures such as doublets and bundles was achieved. Because cleavage of the *C*-terminal tail of tubulin enables the formation of bundles^37^ and B-tubules^21^*in vitro*, the deletion or coverage of *C*-terminal tails is an important factor for forming microtubule superstructures. The binding domains of MAPs to the *C*-terminal tail of tubulin are diverse and include loops, α-helices, β-sheets and β-hairpins, and binding mainly relies on electrostatic interactions.^29^ The electrostatic interaction between positively charged domains of MAPs and the negatively charged *C*-terminal tail of tubulin is expected to neutralize the negatively charged surface of microtubules and reduce electrostatic repulsion between microtubules, resulting in bundling.^15,38^ Although KA7 is a disordered peptide with no specific secondary structure, the positive charge of KA7 was sufficient to facilitate *C*-terminal tail binding.^29^ The multivalent peptide construct consisting of four KA7s was shown to induce the bundled structures by crosslinking microtubules; however, the formation of doublet microtubules was not observed.^29^ Although the bundles can be formed by neutralizing the negatively charged *C*-terminal tail of tubulin or crosslinking of microtubules, it remains challenging to construct doublet microtubules. *In vivo*, it has been suggested that specific MIPs work together to generate doublet microtubules.^17–20^ For example, several proteins, such as CFAP77, firmly fix the newly formed B-tubule hook by binding to the outer junction region of microtubules to stabilize B-tubules.^20^ The inner junction complex proteins such as PACRG and FAP20 also play a role in closing the formed B-tubule.^17,18^ Thus, the formation of doublet microtubules requires the recruitment of free tubulin and the stabilization and closure of B-tubules.

Our previous study proposed that the binding of TP–AG to the *C*-terminal tails on microtubules and subsequent recruitment of tubulin by binding to the TP moiety of TP–AG induced the formation of doublet microtubules.^24^ In this work, we demonstrated that the simple KA7–(GGGS)_3_–TP peptide is sufficient for forming doublet microtubules by binding to the *C*-terminal tails of tubulins of preassembled microtubules by the KA7 moiety and recruitment of new tubulin by the TP moiety. KA7–(GGGS)_3_–TP (4.4 kDa) is a relatively small synthetic peptide compared with TP–AG (118 kDa in the tetrameric form). It is biologically intriguing that the small KA7–(GGGS)_3_–TP without large protein scaffolds can induce the formation of doublet microtubules. However, the doublet microtubules induced by KA7–(GGGS)_3_–TP appeared less stable than those induced by TP–AG or the natural doublet microtubules obtained from cilia and flagella. For example, the detachment of additional layers of the doublet microtubules induced by KA7–(GGGS)_3_–TP (Fig. 5) was not observed in the TEM images of the natural doublet microtubules isolated from *Chlamydomonas* and *Tetrahymena*.^17–20,39^ One of the reasons for the low stability of the doublet microtubules induced by KA7–(GGGS)_3_–TP may be caused by the flexibility of KA7–(GGGS)_3_–TP as a scaffold to fix the recruited tubulin on the surface of preassembled microtubules compared with the protein-based anchoring by TP–AG and *in vivo*. Another possibility is the moderate closing effect of KA7–(GGGS)_3_–TP, as peeled structures and sheets were observed (Fig. 5). The partial detachment of B-tubules from doublet microtubules was observed when specific MIPs were deleted,^17–19^ showing the importance of the fixing and closing effects on the construction of stable doublet microtubules. The peeled structures and sheets were not observed when TP–AG was used,^24^ suggesting the method using KA7–(GGGS)_3_–TP is useful for evaluating the structures of these additional layers. However, using this method to form specific microtubule superstructures, such as doublet microtubules, remains challenging. Combining KA7–(GGGS)_3_–TP with MIPs, which have specific functions such as stabilization and closing, should facilitate the formation of stable doublet microtubules with high selectivity. Further peptide design, including the use of natural binding domains of MAPs/MIPs instead of KA7, multivalent peptides containing multiple TPs and the display of TP on microtubules through covalent bonding, may yield a new approach to forming stable microtubule superstructures. In addition, the lower dynamic instability of axonemal microtubules compared to cytoplasmic microtubules is also presumed to contribute the higher stability of axonemal microtubules.^39^ Evaluating dynamic property will be useful in characterizing the artificially formed doublet microtubules.

## Conclusions

In this report, a simple design of peptides consisting of TP and KA7 enabled the formation of microtubule superstructures such as doublets and bundles by binding of the peptides on the outer surface of microtubules. Notably, displaying the TP moiety on microtubules by the peptide-based approach was sufficient to induce the formation of doublet microtubules. The additional layers of microtubule superstructures induced by KA7–(GGGS)_3_–TP can dissociate, indicating that the peptide-based approach is useful for analyzing the mechanism of formation and dissociation of doublet microtubules when compared with our previous protein-based approach. An additional advantage of the peptide-based approach is that the peptide can be chemically modified. For example, photo-responsive molecules such as photochromic, photocleavable and photocrosslinking molecules can be conjugated to the peptides for photo-controlling the formation and dissociation of microtubule superstructures. Furthermore, the binding properties of peptides to microtubules may be modulated by various approaches such as screening peptide libraries, incorporating unnatural amino acids and computational methods (*e.g.*, simulations, artificial intelligence-based approaches and *de novo* design). Such properties may be useful for controlling the structures and stability of the microtubule superstructures. The peptide-induced formation of microtubule superstructures provides insights into the principles of microtubule superstructure formation and offers a potential approach in nanotechnological applications.

## Materials and methods

### General

RP-HPLC was performed using a Shimadzu LC-6AD liquid chromatograph with GL Science Inertsil WP300 C18 columns (4.6 × 250 mm for analysis and 20 × 250 mm for purification). Fully automated solid-phase peptide synthesis was carried out using a Syro I (Biotage). MALDI-TOF mass spectra were taken using an UltrafleXtreme (Bruker Daltonics) with α-cyano-4-hydroxycinnamic acid (α-CHCA) as a matrix. UV-vis spectra were obtained using a Jasco V-630. Ultracentrifugation was performed using an Optima MAX-TL ultracentrifuge (Beckman Coulter) using a TLA 120.2 rotor. Microtubules were visualized by CLSM using a FluoView FV10i (Olympus) or an epi-fluorescence microscope (Eclipse Ti2-E; Nikon) using an oil-coupled Lambda S 60× objective (NA 1.4) (Nikon). Tubulin was purified from porcine brain by a reported procedure.^40^ AF-tubulin and TMR-tubulin were prepared following a standard protocol by modifying AF and TMR to tubulin, respectively.^41^ The reagents used were purchased from Watanabe Chemical Ind., Ltd, Tokyo Chemical Industry Co., Dojindo Laboratories Co., Ltd, FUJIFILM Wako Pure Chemical Industries, and Sigma-Aldrich. All the reagents were used without further purification.

### Synthesis of peptides

For TMR–KA7–GGGS–TP, TMR–Lys(Boc)–Ala–Lys(Boc)–Ala–Lys(Boc)–Ala–Lys(Boc)–Ala–Lys(Boc)–Ala–Lys(Boc)–Ala–Lys(Boc)–Ala–Gly–Gly–Gly–Ser(Trt)–Gly–Gly–Gly–Lys(Boc)–Lys(Boc)–His(Trt)–Val–Pro–Gly–Gly–Gly–Ser(Trt)–Val–Gln(Trt)–Ile–Val–Tyr(^*t*^Bu)–Lys(Boc)–Pro–Val–Asp(O^*t*^Bu)–Leu–Alko–PEG resin was synthesized on Fmoc–Leu–Alko–PEG resin (Watanabe Chemical Ind. Ltd) using standard Fmoc-based solid phase chemistry using 4 equiv. Fmoc–amino acids. *N*,*N*-Dimethylformamide (DMF) solution of 2-(1*H*-benzotriazole-1-yl)-1,1,3,3-tetramethyluronium hexafluorophosphate (HBTU, 4 equiv.), 1-hydroxybenzotriazole monohydrate (HOBt·H_2_O, 4 equiv.), and diisopropylethylamine (DIPEA, 4 equiv.) were used as condensation reagents. Each condensation reaction was performed using automated peptide synthesizer (Syro I, Biotage). Deprotection of Fmoc groups from the resin was performed using 40% piperidine in DMF. For the introduction of TMR moiety to the *N*-terminal of the peptide, *N*-methylpyrrolidone (NMP) solution of 5-carboxy TMR (4 equiv.), (1-cyano-2-ethoxy-2-oxoethylidenaminooxy) dimethylamino-morpholinocarbenium hexafluorophosphate (COMU, 4 equiv.) and DIPEA (4 equiv.) were added to the resin and stirred at room temperature for 6 h. The peptidyl-resin was washed with NMP, CH_2_Cl_2_, and then dried under vacuum. The peptide was deprotected and cleaved from the resin by treatment with a cleavage cocktail (trifluoroacetic acid (TFA)/water/ethanedithiol/triisopropylsilane = 94/2.5/2.5/1, v/v/v/v). The mixture was kept at room temperature for 3 h. After filtration, the peptide was precipitated by adding ice-cooled *tert*-butyl methyl ether. After centrifugation, the peptide was washed with *tert*-butyl methyl ether 4 times. The precipitated peptide was dried under vacuum. The obtained produce was purified by RP-HPLC with elution of a linear gradient of water/acetonitrile containing 0.1% TFA (95/5 to 0/100, v/v over 95 min, 10 mL min^−1^, detected at 220 nm and 551 nm). MALDI-TOF-MS: *m*/*z* found: 4258 ([M]^+^), calcd 4258.

For TMR–KA7–(GGGS)_3_–TP, TMR–Lys–Ala–Lys–Ala–Lys–Ala–Lys–Ala–Lys–Ala–Lys–Ala–Lys–Ala–Gly–Gly–Gly–Ser–Gly–Gly–Gly–Ser–Gly–Gly–Gly–Ser–Gly–Gly–Gly–Lys–Lys–His–Val–Pro–Gly–Gly–Gly–Ser–Val–Gln–Ile–Val–Tyr–Lys–Pro–Val–Asp–Leu–OH was prepared by the same procedure described above. MALDI-TOF-MS: *m*/*z* found: 4774 ([M]^+^), calcd 4774.

For TMR–KA7–(EAAAK)_2_–TP, TMR–Lys–Ala–Lys–Ala–Lys–Ala–Lys–Ala–Lys–Ala–Lys–Ala–Lys–Ala–Glu–Ala–Ala–Ala–Lys–Glu–Ala–Ala–Ala–Lys–Gly–Gly–Gly–Lys–Lys–His–Val–Pro–Gly–Gly–Gly–Ser–Val–Gln–Ile–Val–Tyr–Lys–Pro–Val–Asp–Leu–OH was prepared by the same procedure described above. MALDI-TOF-MS: *m*/*z* found: 4940 ([M]^+^), calcd 4941.

For KA7–GGGS–TP, H–Lys–Ala–Lys–Ala–Lys–Ala–Lys–Ala–Lys–Ala–Lys–Ala–Lys–Ala–Gly–Gly–Gly–Ser–Gly–Gly–Gly–Lys–Lys–His–Val–Pro–Gly–Gly–Gly–Ser–Val–Gln–Ile–Val–Tyr–Lys–Pro–Val–Asp–Leu–OH was prepared by the same procedure described above. MALDI-TOF-MS: *m*/*z* found: 3846 ([M + H]^+^), calcd 3846.

For KA7–(GGGS)_3_–TP, H–Lys–Ala–Lys–Ala–Lys–Ala–Lys–Ala–Lys–Ala–Lys–Ala–Lys–Ala–Gly–Gly–Gly–Ser–Gly–Gly–Gly–Ser–Gly–Gly–Gly–Ser–Gly–Gly–Gly–Lys–Lys–His–Val–Pro–Gly–Gly–Gly–Ser–Val–Gln–Ile–Val–Tyr–Lys–Pro–Val–Asp–Leu–OH was prepared by the same procedure described above. MALDI-TOF-MS: *m*/*z* found: 4363 ([M + H]^+^), calcd 4363.

For KA7–(EAAAK)_2_–TP, H–Lys–Ala–Lys–Ala–Lys–Ala–Lys–Ala–Lys–Ala–Lys–Ala–Lys–Ala–Glu–Ala–Ala–Ala–Lys–Glu–Ala–Ala–Ala–Lys–Gly–Gly–Gly–Lys–Lys–His–Val–Pro–Gly–Gly–Gly–Ser–Val–Gln–Ile–Val–Tyr–Lys–Pro–Val–Asp–Leu–OH was prepared by the same procedure described above. MALDI-TOF-MS: *m*/*z* found: 4528 ([M]^+^), calcd 4528.

For TMR–KA7, TMR–Lys–Ala–Lys–Ala–Lys–Ala–Lys–Ala–Lys–Ala–Lys–Ala–Lys–Ala–OH was prepared by the same procedure described above. MALDI-TOF-MS: *m*/*z* found: 1826 ([M + H]^+^), calcd 1826.

For KA7, H–Lys–Ala–Lys–Ala–Lys–Ala–Lys–Ala–Lys–Ala–Lys–Ala–Lys–Ala–OH was prepared by the same procedure described above. MALDI-TOF-MS: *m*/*z* found: 1414 ([M + H]^+^), calcd 1414.

For TP, H–Gly–Gly–Gly–Lys–Lys–His–Val–Pro–Gly–Gly–Gly–Ser–Val–Gln–Ile–Val–Tyr–Lys–Pro–Val–Asp–Leu–OH was prepared by the same procedure described above. MALDI-TOF-MS: *m*/*z* found: 2194 ([M + H]^+^), calcd 2194.

### CLSM measurement

The glass bottom dishes (Matsunami, Osaka, Japan) were coated with 1 mg mL^−1^ poly-l-lysine (*M*_w_: 30 000–70 000, Sigma) at room temperature for 1 h, then removed and dried. The microtubule samples were put on the plate and kept at room temperature for ∼1 h, then observed by CLSM. For the flow cell experiment, a flow cell was prepared by making a narrow channel on a 24 mm × 60 mm coverslip covered with 18 mm × 18 mm coverslip (Matsunami, Osaka, Japan) using double-sided tape as a spacer. To the flow cell, 0.5 mg mL^−1^ protein A was applied and incubated for 5 min, then washed by BRB80 (80 mM PIPES pH 6.9, 1.0 mM MgCl_2_, 1.0 mM EGTA) at room temperature. Next, 0.5 mg mL^−1^ anti-β-tubulin, monoclonal antibody solution was applied and incubated for 5 min, then washed by BRB80. Finally, microtubule samples were introduced and incubated for 5 min, then washed by BRB80 and observed by CLSM. AF-tubulin was excited by a 499 nm laser and observed through a 520 nm emission band-pass filter (green). TMR-tubulin and TMR-labeled peptides were excited by a 550 nm laser and observed through a 574 nm emission band-pass filter (red).

### Binding analysis of TMR–KA7–TP to microtubules

To a solution containing tubulin (50 μM, 2 μL) and AF-tubulin (50 μM, 2 μL) in BRB80 and 2 × BRB (160 mM PIPES pH 6.9, 2.0 mM MgCl_2_, 2.0 mM EGTA, 2 μL), GMPCPP premix (1 mM GMPCPP, 20 mM MgCl_2_ in BRB80, 2 μL) was added and incubated at 37 °C for 30 min in the dark. Then TMR–KA7–TP or TMR–KA7 in water (400 μM, 2 μL) was added to the mixture and incubated at 25 °C for 30 min in the dark (final concentrations: [tubulin] = 10 μM, [AF-tubulin] = 10 μM, [TMR–KA7–TP] = 80 μM, [GMPCPP] = 0.2 mM).

Water was added instead of the TMR peptides to prepare the control sample of AF-microtubules without TMR peptides. For the control sample of unlabeled microtubules with TMR–KA7, tubulin (50 μM, 4 μL) was used instead of tubulin (50 μM, 2 μL) and AF-tubulin (50 μM, 2 μL). The mixture was used for CLSM imaging using poly-l-lysine plates. AF and TMR fluorescence intensity per microtubule were measured from the fluorescence images by subtracting the background intensity using ImageJ software (NIH). The background-subtracted TMR fluorescence intensity per AF fluorescence intensity for each microtubule (*N* = 20) was calculated to estimate the binding of TMR-labeled peptides to microtubules.

### Analysis of binding sites of TMR–KA7–TP to microtubules

Microtubules were prepared as above (final concentrations: [tubulin] = 24 μM, [AF-tubulin] = 6 μM, [GMPCPP] = 0.4 mM). Subtilisin (10 μM, 1 μL) was added to the microtubule solution (8 μL) and incubated at 37 °C for 30 min in the dark. Then the subtilisin activity was inhibited by incubation with phenylmethylsulphonyl fluoride (50 mM, 1 μL) at 37 °C for 10 min. After centrifugation of the solution (7 μL) at 18 000 rpm at 37 °C for 20 min, the pellets were resuspended with BRB80. The solution (7 μL) was mixed with TMR–KA7–TP (80 μM, 3 μL) and incubated at 25 °C for 30 min in the dark. The mixture was used for CLSM imaging using poly-l-lysine plates. Water was added instead of subtilisin as a control. AF and TMR fluorescence intensity per microtubule were measured from the fluorescence images by subtracting the background intensity using ImageJ software. The background-subtracted TMR fluorescence intensity per AF fluorescence intensity for each microtubule (*N* = 28) was calculated to estimate the binding of TMR-labeled peptides to microtubules.

### Formation of microtubule superstructures

In the “doublet method”, GMPCPP premix (2 μL) was added to a solution containing tubulin (9 μM, 2 μL) and TMR-tubulin (9 μM, 2 μL) in BRB80 and 2 × BRB (4 μL) and incubated at 37 °C for 30 min in the dark. Then KA7–TP in water (500 μM, 2 μL) was added to the mixture and incubated at 25 °C for 30 min in the dark. Tubulin (18 μM, 2 μL) and AF-tubulin (3.6 μM, 2 μL) in BRB80 were added to the mixture and incubated at 25 °C for 30 min in the dark. Then GMPCPP premix (2 μL) was added and incubated at 37 °C for 30 min in the dark (final concentrations: [tubulin] = 2.7 μM, [TMR-tubulin] = 0.9 μM, [AF-tubulin] = 0.36 μM, [KA7–TP] = 100 μM, [GMPCPP] = 0.2 mM). The mixture was used for CLSM imaging using poly-l-lysine plates or flow cells. Water, KA7, and TP were added instead of KA7–TP as a control. The concentration dependence of KA7–(GGGS)_3_–TP was evaluated using an epi-fluorescence microscope.

In the “singlet method”, TMR-labeled microtubules were prepared and incubated with KA7–(GGGS)_3_–TP same as Double method. AF-labeled microtubules were prepared by incubation of a solution (8 μL) containing tubulin (5.6 μM) and AF-tubulin (1.1 μM) in BRB80 with GMPCPP premix (2 μL) and incubated at 37 °C for 30 min in the dark. The AF-labeled microtubules (4 μL) were mixed with the KA7–(GGGS)_3_–TP-bound TMR-microtubules (14 μL) and incubated at 25 °C for 30 min in the dark. Then GMPCPP premix (2 μL) was added and incubated at 37 °C for 30 min in the dark (Final concentrations: [tubulin] = 2.7 μM, [TMR-tubulin] = 0.9 μM, [AF-tubulin] = 0.36 μM, [KA7–(GGGS)_3_–TP] = 100 μM, [GMPCPP] = 0.2 mM). The mixture was used for CLSM imaging using flow cells. PCC was calculated by the Coloc 2 program in Fiji according to the following equation using the images consisting of red and green channels.
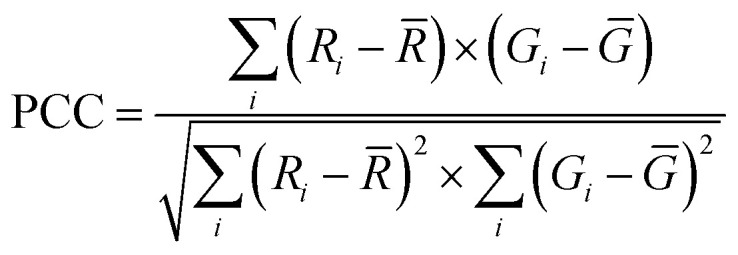
where *R*_*i*_ and *G*_*i*_ are the fluorescence intensity of pixel *i* of the red and green channels, respectively, and *R̄* and *Ḡ* are the mean fluorescence intensity of the red and green channels across the entire image, respectively.

### TEM measurement

Microtubule samples prepared as above were diluted 5-fold with BRB80. The solution (5 μL) was put on a hydrophilized thin carbon-film coated TEM grid (ALLIANCE Biosystems Inc.), allowed to stand for 1.5 min, and then removed. The grid was exposed to 17% EM stainer (Nisshin EM Co., Ltd) (5 μL) for staining, which was allowed to stand for 3.5 min, and then removed. The resulting grid was dried *in vacuo* and observed by TEM (Jeol JEM 1400 Plus) using an accelerating voltage of 80 kV.

### Stability analysis of microtubule superstructures

Microtubule samples were prepared by the doublet or singlet method (final concentrations: [tubulin] = 2.7 μM, [TMR-tubulin] = 0.9 μM, [AF-tubulin] = 0.36 μM, [KA7–(GGGS)_3_–TP] = 100 μM, [GMPCPP] = 0.2 mM). The microtubule samples were incubated at 4 °C for 0–30 min in the dark and used for CLSM imaging using poly-l-lysine plates. In the doublet method, TMR and AF fluorescence intensity per microtubule were measured from the fluorescence images by subtracting the background intensity using ImageJ software. The background-subtracted AF fluorescence intensity per TMR fluorescence intensity for each microtubule (*N* = 12) was calculated to estimate the amount of AF-microtubules on TMR-microtubules. In the singlet method, the background-subtracted average fluorescence intensity of AF-microtubules and the background-subtracted average fluorescence intensity of TMR-microtubules were calculated separately from different microtubules (*N* = 12) because of the lower amount of co-localized TMR-microtubules and AF-microtubules.

## Data availability

All data are provided in the figures and the ESI† figures. Reasonable requests for additional information can be made to the corresponding authors.

## Conflicts of interest

There are no conflicts to declare.

## Supplementary Material

CB-OLF-D4CB00290C-s001
