# Supplementary material for: Peptide-mediated display of Tau-derived peptide for construction of microtubule superstructures
Source: RSC Chem Biol. 2025 Mar 19;6(5):737–45. doi: 10.1039/d4cb00290c (PMC11951922; doi:10.1039/d4cb00290c)
Supplement: CB-006-D4CB00290C-s001 [file CB-006-D4CB00290C-s001.pdf]

Supplementary Information (SI) for RSC Chemical Biology.
This journal is © The Royal Society of Chemistry 2025

Supporting Information

Peptide-mediated display of Tau-derived peptide for construction of microtubule superstructures

Hiroshi Inaba,* Daichi Kageyama, Soei Watari, Mahoko Tateishi, Akira Kakugo, and Kazunori Matsuura*

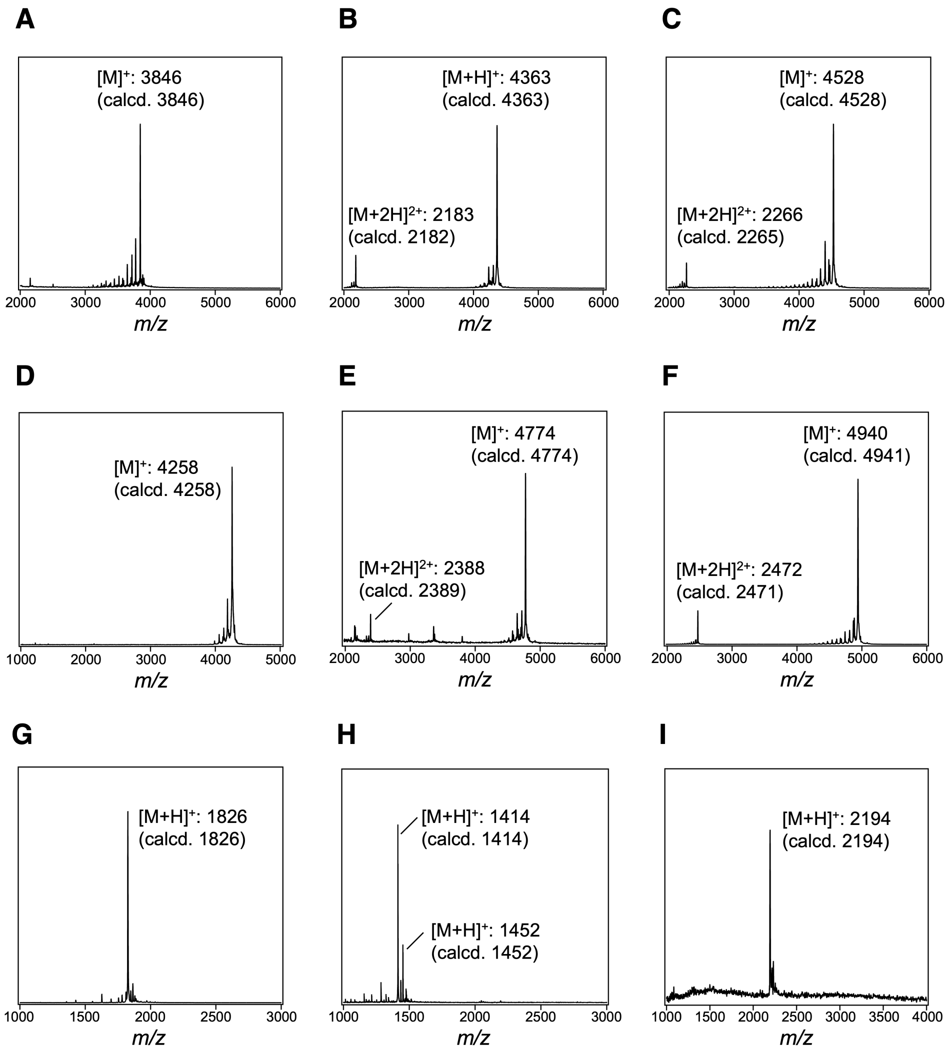

Fig. S1. MALDI-TOF-MS of (A) KA7-GGGS-TP, (B) KA7-$(GGGS)_3$-TP, (C) KA7-$(EAAAK)_2$-TP, (D) TMR-KA7-GGGS-TP, (E) TMR-KA7-$(GGGS)_3$-TP, (F) TMR-KA7-$(EAAAK)_2$-TP, (G) TMR-KA7, (H) KA7, (I) TP.

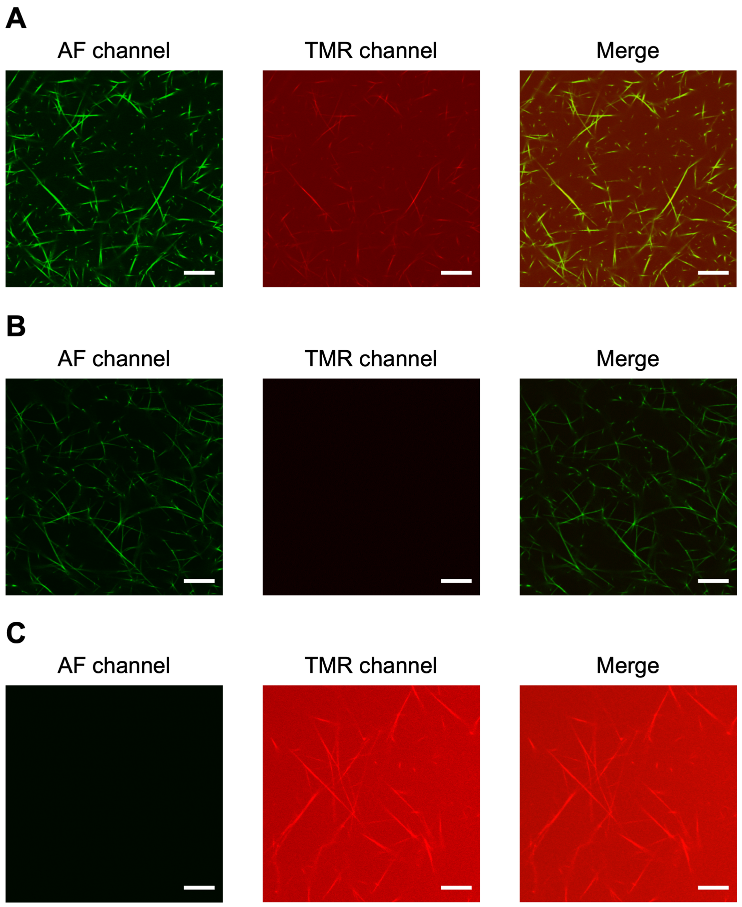

Fig. S2. CLSM images of (A) AF-microtubules bound with TMR-KA7, (B) AF-microtubules without TMR-peptides, and (C) unlabeled microtubules bound with TMR-KA7. Preparation concentrations: [Tubulin] = 10 μM; [AF-tubulin] = 10 μM; [TMR-KA7] = 80 μM; [GMPCPP] = 0.2 mM for (A), [Tubulin] = 10 μM; [AF-tubulin] = 10 μM; [GMPCPP] = 0.2 mM for (B), [Tubulin] = 20 μM; [TMR-KA7] = 80 μM; [GMPCPP] = 0.2 mM for (C). Scale bars, 10 μm.

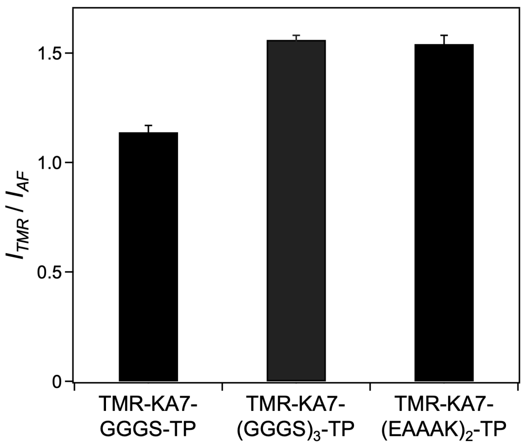

Fig. S3. The I_{TMR}/I_{AF} ratio showing TMR-KA7-TP fluorescence per microtubule determined from the CLSM images (Fig. 2B). Error bars represent the standard error of the mean (N = 20).

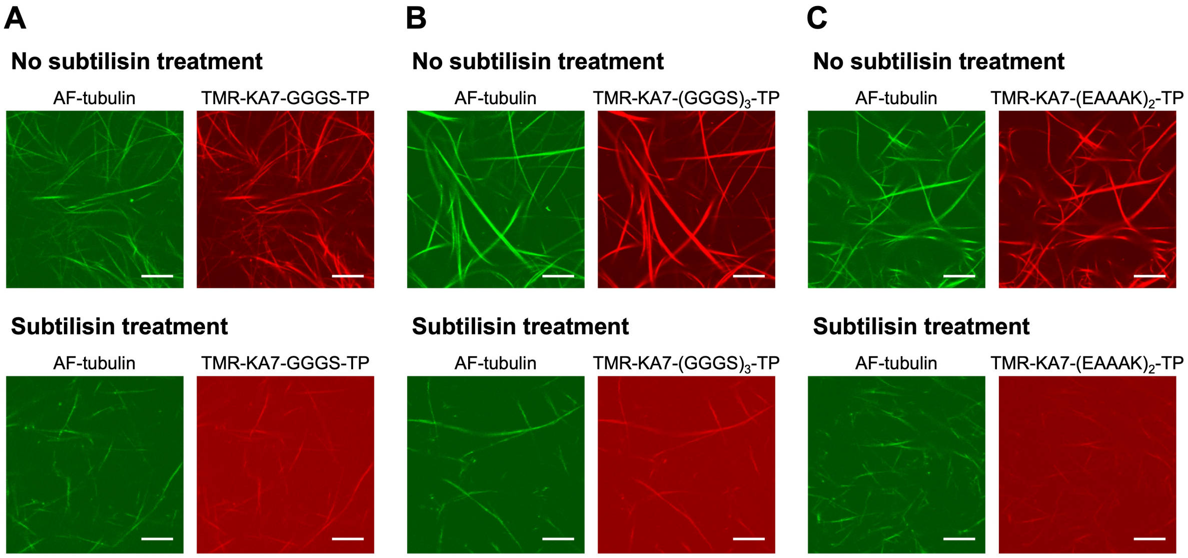

Fig. S4. Representative CLSM images of microtubules bound with (A) TMR-KA7-GGGS-TP, (B) TMR-KA7-$(GGGS)_3$-TP, and (C) TMR-KA7-$(EAAAK)_2$-TP with and without subtilisin treatment. Microtubules were prepared, treated with or without subtilisin, and then incubated with TMR-KA7-TP. Preparation concentrations: [Tubulin] = 13.4 μM; [AF-tubulin] = 3.4 μM; [TMR-KA7-TP] = 24 μM; [Subtilisin] = 0.7 μM; [GMPCPP] = 0.14 mM.

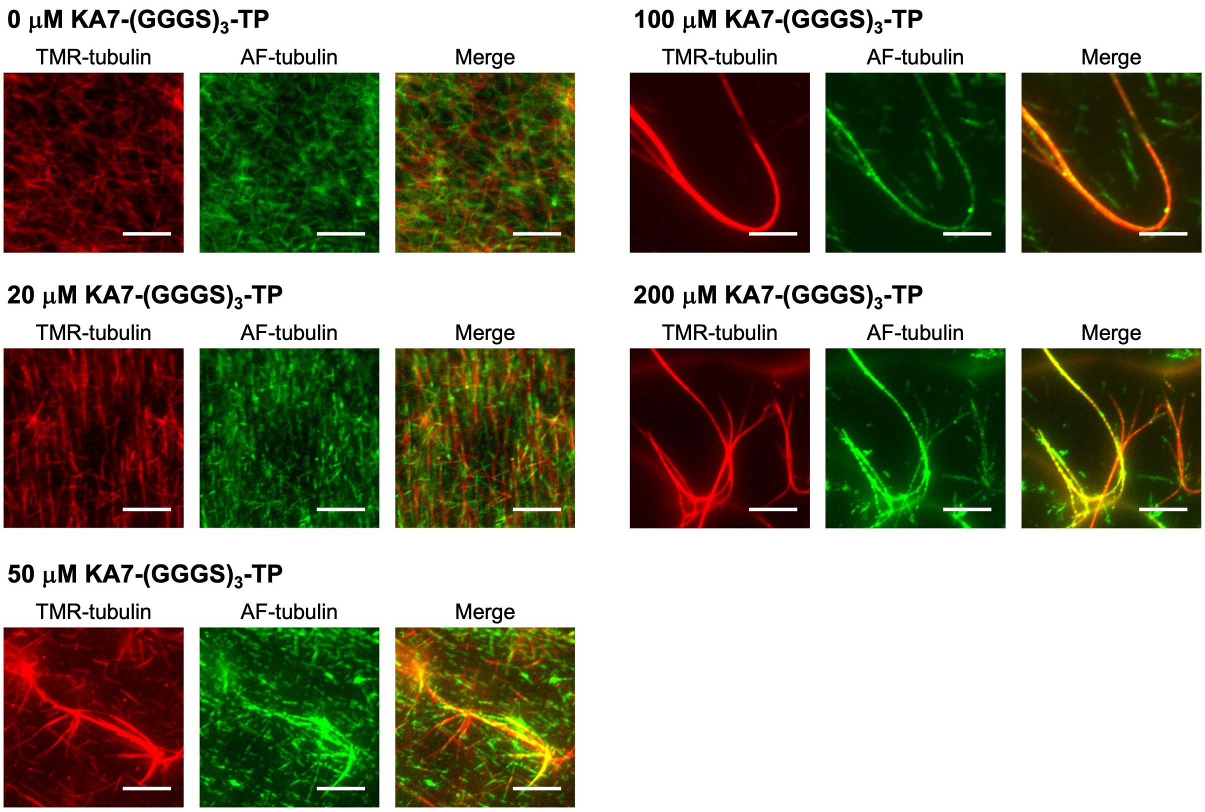

Fig. S5. Concentration dependence of KA7-(GGGS)$_3$-TP on the formation of microtubule superstructures as shown in Fig. 3. Preparation concentrations: [tubulin] = 2.7 μM; [TMR-tubulin] = 0.9 μM; [AF-tubulin] = 0.36 μM; [KA7-(GGGS)$_3$-TP] = 0–200 μM; [GMPCPP] = 0.2 mM. Scale bars, 10 μm.

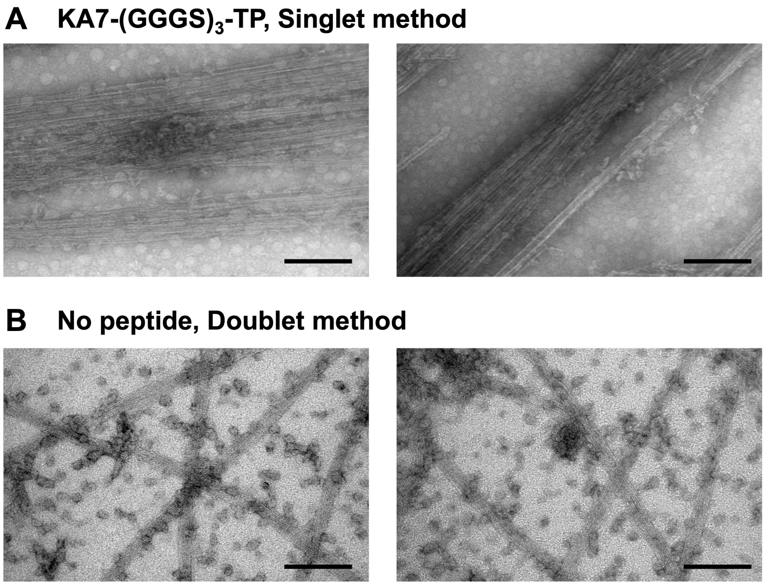

Fig. S6. TEM images of microtubules (A) prepared using KA7-(GGGS)$_3$-TP by the Singlet method and (B) prepared without peptides by the Doublet method. Preparation concentrations: [tubulin] = 2.7 μM; [TMR-tubulin] = 0.9 μM; [AF-tubulin] = 0.36 μM; [KA7-(GGGS)$_3$-TP] = 100 μM; [GMPCPP] = 0.2 mM. Scale bars, 200 nm.

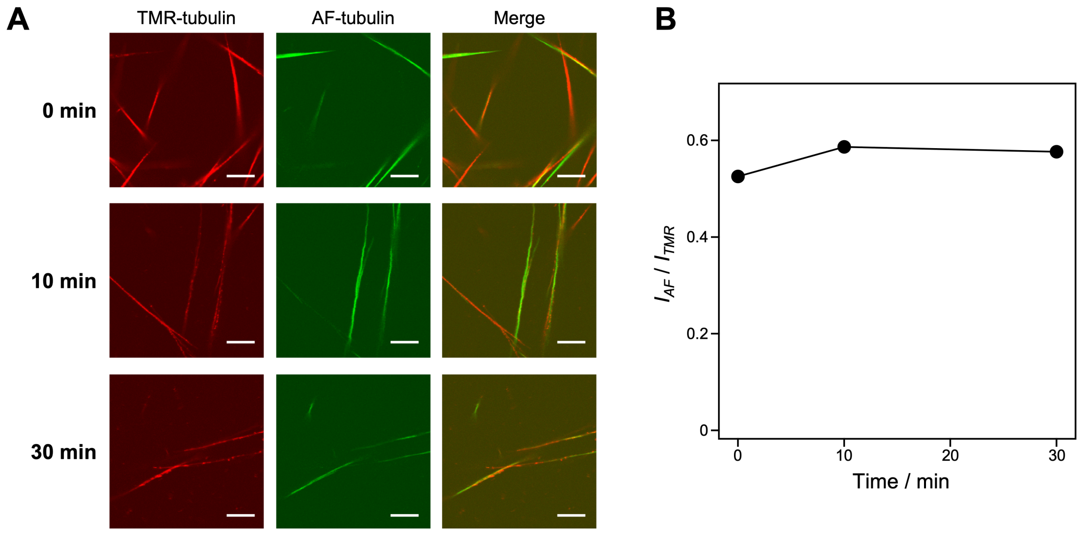

Fig. S7. (A) CLSM images of microtubule superstructures induced by KA7-(GGGS)$_3$-TP using the Singlet method, keeping at 4 °C for 0–30 min. Preparation concentrations: [Tubulin] = 2.7 μM; [TMR-tubulin] = 0.9 μM; [AF-tubulin] = 0.36 μM; [KA7-(GGGS)$_3$-TP] = 100 μM; [GMPCPP] = 0.2 mM. Scale bars, 10 μm. (B) The I_{AF}/I_{TMR} ratio, the average fluorescence intensity of AF-microtubules per the average fluorescence intensity of TMR-microtubules separately determined from the CLSM images (N = 12).
